# Willingness to pay for community delivery of antiretroviral treatment in urban Tanzania: a cross-sectional survey

**DOI:** 10.1093/heapol/czaa088

**Published:** 2020-10-23

**Authors:** Pascal Geldsetzer, Alexander Sauer, Joel M Francis, Eric Mboggo, Sharon Lwezaula, David Sando, Wafaie Fawzi, Nzovu Ulenga, Till Bärnighausen

**Affiliations:** 1 Division of Primary Care and Population Health, Department of Medicine, Stanford University School of Medicine, 1265 Welch Road, Stanford, CA 94305, USA; 2 Heidelberg Institute of Global Health (HIGH), Heidelberg University, Im Neuenheimer Feld 130.3, 69120 Heidelberg, Germany; 3 Department of Statistics, University of Oxford, 24-29 St Giles', Oxford OX1 3LB, UK; 4 Department of Family Medicine and Primary Care, School of Clinical Medicine, University of the Witwatersrand, Johannesburg, Gauteng, South Africa; 5 Management and Development for Health, Plot #802, Mwai Kibaki Road, Mikocheni, Dar es Salaam, Tanzania; 6 National AIDS Control Program, Lithuli Street, Dar es Salaam, P.O. Box 11857, Tanzania; 7 Department of Global Health and Population, Harvard T.H. Chan School of Public Health, Boston, MA, USA; 8 Department of Epidemiology, Harvard T.H. Chan School of Public Health, 677 Huntington Avenue, Boston, MA 02115, USA; 9 Department of Nutrition, Harvard T.H. Chan School of Public Health, 677 Huntington Avenue, Boston, MA 02115, USA; 10 Africa Health Research Institute (AHRI), Africa Centre Building, Via R618 to Hlabisa, Somkhele, P.O. Box 198, Mtubatuba 3935, South Africa

**Keywords:** Community health worker, differentiated antiretroviral therapy, HIV, willingness to pay, healthcare financing, Tanzania

## Abstract

Community health worker (CHW)-led community delivery of HIV antiretroviral therapy (ART) could increase ART coverage and decongest healthcare facilities. It is unknown how much patients would be willing to pay to receive ART at home and, thus, whether ART community delivery could be self-financing. Set in Dar es Salaam, this study aimed to determine patients’ willingness to pay (WTP) for CHW-led ART community delivery. We sampled ART patients living in the neighbourhoods surrounding each of 48 public-sector healthcare facilities in Dar es Salaam. We asked participants (*N* = 1799) whether they (1) preferred ART community delivery over standard facility-based care, (2) would be willing to pay for ART community delivery and (3) would be willing to pay each of an incrementally increasing range of prices for the service. 45.0% (810/1799; 95% CI: 42.7—47.3) of participants preferred ART community delivery over standard facility-based care and 51.5% (417/810; 95% CI: 48.1—55.0) of these respondents were willing to pay for ART community delivery. Among those willing to pay, the mean and median amount that participants were willing to pay for one ART community delivery that provides a 2-months’ supply of antiretroviral drugs was 3.61 purchasing-power-parity-adjusted dollars (PPP$) (95% CI: 2.96–4.26) and 1.27 PPP$ (IQR: 1.27–2.12), respectively. An important limitation of this study is that participants all resided in neighbourhoods within the catchment area of the healthcare facility at which they were interviewed and, thus, may incur less costs to attend standard facility-based ART care than other ART patients in Dar es Salaam. While there appears to be a substantial WTP, patient payments would only constitute a minority of the costs of implementing ART community delivery. Thus, major co-financing from governments or donors would likely be required.

**Key Messages**

- Thus far, there have been no studies of willingness to pay for a chronic disease care service provided by community health workers in a low- or middle-income country.
- In the context of plateauing or decreasing donor funds for HIV care, this study provides important willingness-to-pay data for an HIV care model that could both decongest healthcare facilities and improve antiretroviral therapy (ART) care retention.
- While the funds that could be raised from patients for ART community delivery are considerable, they would only cover a minority of the costs of implementing such a programme.

## Introduction

Largely as a result of the success of antiretroviral therapy (ART) in reducing HIV-related mortality (Hontelez *et al.*, 2012), the number of people living with HIV (PLHIV) globally has been steadily increasing from an estimated 28.0 million in 2000 to 38.8 million in 2015 (Wang *et al.*, 2016). Approximately three-quarters (76%) of the PLHIV globally reside in Sub-Saharan Africa, and less than half (42.4%) of them are estimated to be on ART (Wang *et al.*, 2016). The number of ART patients in Sub-Saharan Africa is projected to increase substantially over the coming years as a result of the rising number of PLHIV and the expansion of ART eligibility (Wang *et al.*, 2016; World Health Organization, 2016).

The expected rise in demand for ART has led to calls for models of ART care that reduce patient volumes at healthcare facilities without adversely affecting quality of care (Lazarus *et al.*, 2014; Phillips *et al.*, 2015; Geldsetzer *et al.*, 2016; Waldrop *et al.*, 2016; World Health Organization, 2016). One care model that could plausibly achieve this aim is community health worker (CHW)-led delivery of ART to patients’ homes and other meeting points in the community (henceforth referred to as ART community delivery). By reducing the frequency with which patients need to attend the healthcare facility for antiretroviral drug refills, CHW-led ART community delivery has the potential to increase retention in ART care, reduce patients’ out-of-pocket expenditures and time lost from work to attend ART care, and increase the quality of facility-based care by decongesting ART clinics. In a recent cluster-randomized trial embedded in the routine health system in Dar es Salaam, we have shown that ART community delivery resulted in a non-inferior health outcome (as measured by virological suppression) compared with standard facility-based care (Geldsetzer *et al.*, 2017, 2018b). Similarly, two further randomized trials of ART community delivery in Sub-Saharan Africa—one conducted in rural Uganda (Jaffar *et al.*, 2009) and one in rural Kenya (Selke *et al.*, 2010)—provide additional evidence in support of CHW-led ART community delivery.

Even in countries in which ART community delivery is implemented by an already-existing CHW cadre, the introduction of this new healthcare delivery model will likely incur costs for additional CHW training, as well as compensation for CHWs’ additional workload and transport costs. Covering these costs would require an investment by the government or donors because any savings to the health system from ART community delivery would likely result in higher productivity from existing resources rather than a reduction in healthcare expenses to the government (Di Giorgio *et al*., 2016; Geldsetzer *et al.*, 2016). Specifically, ART community delivery may reduce total costs to the health system because care is shifted from more highly paid healthcare worker cadres (nurses and physicians) to a less highly paid cadre (CHWs). The costs saved (through reduced salary expenses for nurses and physicians) for ART care could be invested into other healthcare areas, such as by nurses and physicians spending more time than they would otherwise providing non-communicable disease care. However, these savings would only result in reduced expenses in the government budget for health if the government lays off nurses or physicians in response to the ART community delivery programme, which would be undesirable (given the severe shortages in human resources for health in Sub-Saharan Africa; World Health Organisation, 2018) and may also be politically infeasible. Thus, in the face of plateauing or decreasing donor investments into HIV care (Global Burden of Disease Health Financing Collaborator Network, 2018), a crucial question for the scale-up of ART community delivery is how such programmes could be financed.

Many ART patients incur substantial costs from ART care attendance, particularly from transport expenses and lost time from work (Barennes *et al.*, 2015; Chimbindi *et al.*, 2015; Etiaba *et al.*, 2016). ART community delivery would likely lower ART patients’ expenses by reducing the frequency with which patients have to attend the healthcare facility for antiretroviral drug refills. These patients may, therefore, be willing to pay for ART community delivery, which would be a particularly attractive financing option because it does not require governments to raise additional funds and would not be vulnerable to political changes. Using cross-sectional data from a survey among ART patients in Dar es Salaam, Tanzania, this study aimed to determine (1) whether and how much ART patients are willing to pay for ART community delivery and (2) willingness to pay (WTP) varies by socio-demographic and clinical characteristics, which is an important consideration in deciding how much different patients should pay for the service.

## Methods

### Study setting

This study took place at 48 public-sector healthcare facilities across all municipalities of Dar es Salaam, Tanzania. Tanzania is thought to have an HIV prevalence of around 5% among adults aged 15–49 years (UNAIDS, 2019). Dar es Salaam is a highly urbanized region in East Tanzania and the most populous city in Eastern Africa (United Nations, 2016). With the exception of two large hospitals (which were excluded due to an ongoing clinical trial among HIV patients), the study facilities consisted of all facilities in Dar es Salaam that had an affiliated team of public-sector CHWs. They included 3 hospitals, 8 health centres and 37 dispensaries. All public-sector healthcare facilities in Tanzania provide ART services to patients at no cost, including the antiretroviral drugs.

### Selection of survey participants

This study took place from 1 March 2017 to 27 October 2017. The study was embedded in the study exit assessment of a cluster-randomized trial of CHW-led ART community delivery, which has been published separately (Geldsetzer *et al.*, 2018b). In the trial, ART patients in the 24 intervention clusters (a healthcare facility with its catchment area) were eligible for CHW-led ART community delivery if they were clinically stable on ART, which was defined as (1) having taken antiretroviral drugs for at least 6 months prior to study enrolment, (2) having had a CD4 cell count >350 cells/μl or a suppressed viral load at six or more months after ART initiation and (3) the most recent viral load was taken <12 months prior to study enrolment and showed virological suppression.

A study team member was present at each healthcare facility during the trial exit period (Temeke district: on each workday between 1 March 2017 to 31 April 2017 and 1 day a week thereafter; Kinondoni district: on each workday between 1 May 2017 to 30 June 2017 and 1 day a week thereafter; Ilala district: on each workday between 1 July 2017 to 31 August 2017 and 1 day a week thereafter). Study team members interviewed trial participants and ART patients who visited the healthcare facility on one of these data collection days. ART patients who were eligible for the WTP study, but were not participants in the cluster-randomized trial, were sampled by selecting the next patient entering the consultation room. That is, when an interviewer arrived at the healthcare facility or returned from a previous interview, the interviewer would select the next patient who entered the consultation room as opposed to the next patient exiting the consultation room, because the latter tends to result in a sample of participants in which those patients with longer consultation are overrepresented (Geldsetzer *et al.*, 2018a). Participants for the cluster-randomized trial were selected at the same healthcare facilities during the trial enrolment period (Temeke district: 1 March 2016 to 29 July 2016; Kinondoni district: 1 August 2016 to 31 October 2016; Ilala district: 1 November 2016 to 31 January 2017). During this trial enrolment period, the ART nurse at each of the healthcare facilities enquired from all ART patients prior to or during the consultation whether they resided within one of the neighbourhoods that form part of the healthcare facility’s official catchment area. Participants who reported to be living in the catchment area were then referred to the interviewer for the written informed consent procedure.

The study team member introduced the WTP study to the selected patient, and, after obtaining verbal consent, guided the patient to a private area in (or adjacent to) the healthcare facility. The study was then introduced in more depth and all patients signed an informed consent form prior to the interview. The inclusion criteria both for enrolling into the cluster-randomized trial and for participating in the WTP study only were (1) age ≥18 years, (2) having attended one of the participating healthcare facilities for ART care during the enrolment period of the trial or the study period for the WTP study and (3) residing in the facility’s catchment area. Pregnant women were not invited to participate in the study because pregnant women living with HIV were seen in a different section of the healthcare facility at most facilities. We henceforth refer to all respondents in the WTP study as participants, regardless of whether they were participants in the cluster-randomized trial or not.

### Tanzania’s home-based carer programme

This study asked about participants’ WTP to pay for ART delivered by one specific CHW cadre, namely Tanzania’s home-based carer (HBCs). Having existed since 1996, the HBCs are a long-standing national CHW cadre, which is part of Tanzania’s public-sector health system. Each HBC is affiliated with a specific healthcare facility where their supervisor (a nurse) is based. There were ∼35 000 HBCs in Tanzania at the time of the study who were each paid a monthly stipend of 80 000 Tanzanian Shilling [circa US$37 or 112 purchasing-power-parity-adjusted dollars (PPP$)]. HBCs work in the neighbourhoods in which they live, with neighbourhoods having one to three HBCs. HBCs in Dar es Salaam, and Tanzania more broadly, conduct household visits at least every 3 months to HIV patients in their assigned neighbourhood. Their activities during these household visits are the provision of counselling on adherence to ART, family planning, nutrition, promoting the uptake of preventive healthcare services and referring ill clients to a healthcare facility.

### Eliciting WTP for ART community delivery

We used a tablet-based format of the ‘payment card’ format to elicit participants’ WTP, which is a method that was first developed by Mitchell and Carson >30 years ago and has since then been widely used in health-related research (Mitchell and Carson, 1984; Ryan *et al.*, 2001). Specifically, participants were first asked whether they prefer (1) ART community delivery through HBCs every 2 months with a facility-based check-up every 6 months or (2) attending the healthcare facility every 2 months (which is the standard for most ART patients in Dar es Salaam) without any ART community delivery. Those who preferred the first option were then asked whether they would be willing to pay (regardless of how small the amount is) their HBC to receive ART community delivery. Those who were willing to pay some amount were then asked to respond with ‘yes’ or ‘no’ to a given payment range whereby the range increased incrementally and the interviewer was instructed to stop whenever the participant first said ‘no’ to a payment range. The payment range in our questionnaire started with the category of 1–10 Tanzanian Shillings (TSh) because 1 TSh is the lowest possible value and any value below 10 TSh would not result in meaningful revenue generation. The highest payment range to which the participant responded with ‘yes’ was considered the maximum amount that the participant was willing to pay. The exact questions asked to participants to elicit WTP are shown in Supplementary Text S1. Interviewers administered the questionnaire in Swahili using tablets.

Compared with the two main alternative methods to assess WTP, the ‘take-it-or-leave-it’ approach and a bidding format, the payment card method does not suffer from a starting-point bias and is less vulnerable than the ‘take-it-or-leave-it’ approach to yes-saying bias (Mitchell and Carson, 1993). While responses to the payment card method could be influenced by the maximum monetary value in the shown range, there is little empirical evidence for the existence of this possible bias (Klose, 1999; Ryan *et al.*, 2004).

### Statistical analysis

It is likely that in most settings, ART community delivery will only be provided to those who prefer this service over the standard of care. Our WTP calculations, thus, focussed on those participants who stated that they prefer ART community delivery over standard facility-based ART care. This study has three outcomes: (1) whether a participant preferred ART community delivery over standard facility-based care, (2) among patients who preferred ART community delivery, whether the participant was willing to pay for the programme and (3) among participants who were willing to pay, what was the maximum amount that the participant was willing to pay. We calculated the proportions for the first and second outcome. For the third outcome, we computed both the mean and median amount that participants were willing to pay. We also computed the price at which the maximum revenue would be generated by multiplying the proportion of participants who were willing to pay the given price range by the mean of the price range.

Lastly, to investigate which patient characteristics predict each of the outcomes, we regressed each outcome onto patients’ characteristics using one patient characteristic at a time (with results from a covariate-adjusted regression that includes all patient characteristics as independent variables shown in the Supplementary Appendix). For the first and second outcomes, which were both binary, we used Poisson regression with a robust error structure (Zou, 2004). We used Poisson rather than logistic regression because it is also a valid model for binary outcome data but the resulting risk ratio (RR) is more intuitively interpretable than an odds ratio (Gallis and Turner, 2019). For the third outcome, we log-transformed the monetary amount and then regressed it onto patients’ characteristics using ordinary least squares regressions. We multiplied the coefficient of the ordinary least squares regression by 100 so that it can be interpreted as an approximation of the relative change in per cent of the WTP amount as the independent variable increases by one unit. Our primary analysis used covariate-unadjusted models because the aim of our regressions was to examine whether a given patient characteristic predicts the outcome rather than to arrive at causal statements regarding the relationship between the patient characteristic and the outcome. In all regressions, we adjusted standard errors for clustering (using the sandwich estimator) at the level of the healthcare facility.

All analyses were conducted among the sample that included both those survey participants who were also part of the cluster-randomized trial and those who were not. To investigate whether those who have received ART community delivery as part of the trial have a different WTP than those who did not, we included a binary indicator variable for having received ART community delivery in our regression analyses.

All monetary amounts were converted to PPP$ using the PPP conversion factor for the year of data collection (2017). R version 3.5.2 was used for the analyses.

## Results

### Sample characteristics

In total, 1799 ART patients were interviewed (Table 1). Most participants (1464/1799) were women and 74.2% (1336/1799) were aged between 29 and 48 years. Around three-quarters (1418/1799) had attended primary school. Those preferring ART community delivery had similar characteristics as all participants with the exception of a higher proportion who received ART community delivery as part of the cluster-randomized trial [44.7% (362/810) among those preferring ART community delivery vs 21.6% (388/1799) among all participants].

**Table 1:** Sample characteristics

	All participants, *n* (%)	Preferring ART community delivery, *n* (%)
*N*	1799	810
Female^a^^,b^	1464 (81.4)	643 (79.4)
Age (years)^a^		
18–28	153 (8.5)	59 (7.3)
29–38	651 (36.2)	273 (33.7)
39–48	685 (38.1)	322 (39.8)
>48	304 (16.9)	154 (19)
Highest level of school attended		
No schooling or preschool	87 (4.8)	57 (7)
Primary school	1418 (78.8)	638 (78.8)
Secondary school or above	285 (15.8)	113 (14)
Missing	9 (0.5)	2 (0.2)
Currently married^b^	755 (42.0)	354 (43.7)
Missing	21 (1.2)	4 (0.5)
Disclosed HIV status to at least one person^b^	1630 (90.6)	757 (93.5)
Missing	4 (0.2)	1 (0.1)
Received ART community delivery^a^^,b^	388 (21.6)	362 (44.7)
Mode of antiretroviral drug refills		
Once a month at the facility	474 (26.3)	137 (16.9)
Every 2 months at the facility	1200 (66.7)	564 (69.6)
Brought to the patient by an HBC^c^	102 (5.7)	100 (12.3)
Missing	23 (1.3)	9 (1.1)
Total cost for today’s ART visit (PPP$)^a^		
0.00	661 (36.7)	341 (42.1)
0.01–1.00	602 (33.5)	276 (34.1)
1.01–2.00	367 (20.4)	120 (14.8)
>2.00	169 (9.4)	73 (9.0)
Waiting time for today’s ART visit (min)		
0	225 (12.5)	104 (12.8)
1–20	780 (43.4)	304 (37.5)
21–60	465 (25.8)	189 (23.3)
>60	271 (15.1)	179 (22.1)
Missing	58 (3.2)	34 (4.2)
Travel time to the ART clinic (min)		
0–15	600 (33.4)	281 (34.7)
16–30	817 (45.4)	361 (44.6)
31–60	285 (15.8)	117 (14.4)
>60	93 (5.2)	49 (6.0)
Missing	4 (0.2)	2 (0.2)
Number of HBC visits during the study period^a^		
0	1400 (77.8)	450 (55.6)
1–3	205 (11.4)	171 (21.1)
>3	194 (10.8)	189 (23.3)
Years since initiation of ART		
0–3	747 (41.5)	286 (35.3)
4–5	467 (26.0)	222 (27.4)
>5	489 (27.2)	248 (30.6)
Missing	96 (5.3)	54 (6.7)

a No observations were missing for these variables.

b These are binary variables. We show the number and per cent for participants who answered ‘yes’ to this variable (i.e. those who were female rather than male, those who were currently married rather than currently not married, those who disclosed their HIV status to at least one person rather than those who did not disclose their status to anyone, and those who received ART community delivery rather than those who did not receive ART community delivery).

c We suspect that fewer participants answered that they received their ARVs at home through an HBC for this question than for the question on having received ART community delivery because they misunderstood the question to refer to the frequency of antiretroviral drug refills rather than the mode of the refills (picking up antiretroviral drugs at the healthcare facility vs receiving them at home).

PPP$, purchasing-power-parity-adjusted dollars; ART, antiretroviral therapy; HBC, home-based carer.

### WTP for one community delivery of a 2-months’ supply of antiretroviral drugs

45.0% (810/1799) of participants preferred ART community delivery over standard facility-based care. Among these 810 participants, 51.5% (417/810) were willing to pay for ART community delivery. Among those willing to pay, the mean and median amount that participants were willing to pay for one ART community delivery (delivering a 2-months’ supply of antiretroviral drugs) was PPP$3.61 and PPP$1.27, respectively. Among those preferring ART community delivery and all ART patients who were interviewed, the mean WTP was PPP$1.86 and PPP$0.84, respectively. WTP for each patient group (all participants, those preferring ART community delivery, and those willing to pay for ART community delivery) is shown in Supplementary Table S1.

**Figure 1:**
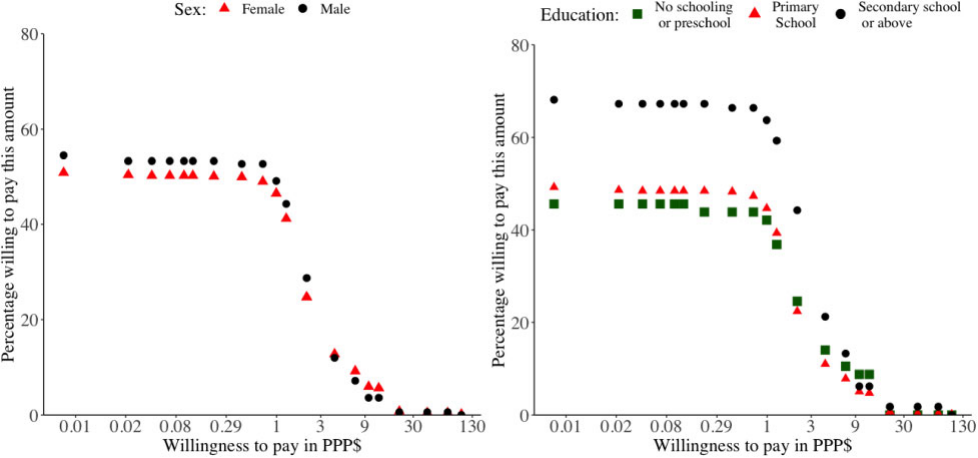
WTP for one community delivery of a 2-months’ supply of antiretroviral drugs among participants preferring ART community delivery over standard facility-based care (*n* = 810). The *x*-axis is on a logarithmic scale. PPP$, purchasing-power-parity-adjusted dollars.

Figure 1 shows that, among those preferring ART community delivery, approximately half of participants were willing to pay all amounts up until the range of PPP$0.57 to PPP$0.85, after which the proportion willing to pay higher amounts decreased rapidly. Virtually all participants’ maximum WTP was reached at the payment range of PPP$14.12 to PPP$28.23. The differences in WTP between men and women were small. However, those who attended secondary school or tertiary education had a markedly higher WTP up until the payment range of 2.82–5.65 PPP$ than those with lower educational attainment. Supplementary Figure S1 disaggregates WTP by whether participants had ever received antiretroviral drugs at home through an HBC.

**Figure 2:**
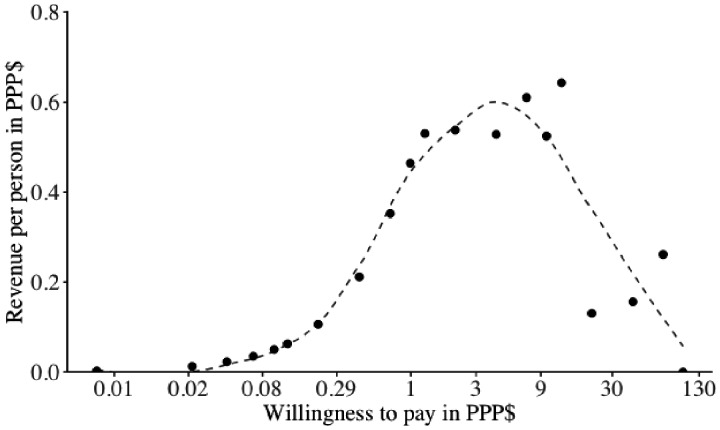
Total revenue generated per person by one community delivery of a 2-months’ supply of antiretroviral drugs. This figure refers to total revenue per person among those participants who preferred ART community delivery over standard facility-based care. The *x*-axis is on a logarithmic scale. The dashed line represents a LOESS regression with a bandwidth of 0.5. PPP$, purchasing-power-parity-adjusted dollars.

If everyone was charged the same price, the maximum revenue for the programme would have been raised at a price of PPP$11.29 to PPP$14.12 for one community delivery of a 2-months’ supply of ART (Figure 2). Taking the mean of that price range (i.e. PPP$12.71), the total revenue generated under this price scheme for the 810 participants who preferred ART community delivery over standard facility-based care would have been PPP$520.70 (or PPP$0.64 per person). However, assuming those not willing to pay this price do not receive the service, the programme would only reach 5.1% (41/810) of participants preferring ART community delivery over standard facility-based care. If everyone was instead charged the maximum amount that they indicated they were willing to pay, then the total revenue for one community delivery of a 2-months’ supply of antiretroviral drugs for these 810 participants would have been PPP$1506.68 (or PPP$1.86 per person).

### Predictors of WTP for ART community delivery

Older individuals were more likely to prefer ART community delivery (Table 2), but less likely to be willing to pay for the service if they did prefer the service. In contrast, those with higher education were less likely to prefer ART community delivery but more likely to be willing to pay for the service if they did prefer the programme over standard facility-based care. Those who had received ART community delivery during the period of the cluster-randomized trial were more likely to prefer ART community delivery but there was no significant difference in the WTP among those who preferred ART community delivery between those who did and did not receive ART community delivery before. Not having disclosed one’s HIV status to at least one person was associated with a lower probability of preferring ART community delivery and, among those who preferred the programme, a lower probability of being willing to pay for the service. The results were similar when running covariate-adjusted instead of unadjusted regressions (Supplementary Table S2).

**Table 2:** Predictors of WTP and preferring ART community delivery over standard facility-based care

	Preferring ART community delivery^a^		Willing to pay for ART community delivery^b^		Amount willing to pay^c^	
	RR (95% CI)	*P*	RR (95% CI)	*P*	% change (95% CI)	*P*
Sex						
Female	1.00 (Ref)		1.00 (Ref)		0.00 (Ref)	
Male	1.14 (0.97–1.33)	0.122	1.07 (0.88–1.30)	0.506	−8.44 (−38.81–21.94)	0.586
Age (years)						
[18,28]	1.00 (Ref)		1.00 (Ref)		0.00 (Ref)	
(28,38]	1.09 (0.88–1.34)	0.433	0.98 (0.78–1.24)	0.895	−15.49 (−51.32–20.33)	0.397
(38,48]	1.22 (0.93–1.59)	0.144	0.78 (0.62–0.99)	0.042	−15.78 (−45.71–14.14)	0.301
>48	1.31 (0.97–1.77)	0.074	0.69 (0.51–0.93)	0.015	−33.65 (−77.38–10.09)	0.132
Education						
None	1.00 (Ref)		1.00 (Ref)		0.00 (Ref)	
Primary school	0.69 (0.56–0.85)	<0.001	1.08 (0.77–1.53)	0.651	−21.14 (−74.38–32.10)	0.436
Secondary school or above	0.61 (0.43–0.86)	0.005	1.49 (1.00–2.22)	0.048	3.99 (−52.61–60.59)	0.890
Marital status						
Not married	1.00 (Ref)		1.00 (Ref)		0.00 (Ref)	
Married	1.06 (0.93–1.21)	0.370	1.19 (1.06–1.33)	0.003	4.87 (−24.41–34.14)	0.745
Years since initiation of ART						
[0,3]	1.00 (Ref)		1.00 (Ref)		0.00 (Ref)	
(3,5]	1.24 (1.04–1.49)	0.018	0.98 (0.81–1.19)	0.844	21.46 (−3.46–46.37)	0.091
>5	1.32 (1.02–1.72)	0.036	1.07 (0.85–1.35)	0.573	−10.79 (−36.90–15.33)	0.418
Mode of ART provision						
Once a month	1.00 (Ref)		1.00 (Ref)		0.00 (Ref)	
Every 2 months	1.63 (1.18–2.24)	0.003	1.22 (0.92–1.61)	0.160	−49.68 (−120.38–21.03)	0.168
Brought home	3.39 (2.53–4.55)	<0.001	1.21 (0.75–1.94)	0.429	−61.70 (−134.42–11.02)	0.096
Disclosed HIV status						
Yes	1.00 (Ref)		1.00 (Ref)		0.00 (Ref)	
No	0.68 (0.52–0.89)	0.005	0.66 (0.43–1.00)	0.049	20.79 (−10.07–51.65)	0.187
Received ART community delivery						
No	1.00 (Ref)		1.00 (Ref)		0.00 (Ref)	
Yes	2.94 (2.21–3.91)	<0.001	0.90 (0.69–1.17)	0.424	−28.77 (−72.36–14.82)	0.196
Total costs for today’s ART visit (PPP$)						
0	1.00 (Ref)		1.00 (Ref)		0.00 (Ref)	
(0,1]	0.89 (0.61–1.30)	0.545	1.27 (1.00–1.61)	0.050	26.99 (−2.66–56.64)	0.074
(1,2]	0.63 (0.45–0.89)	0.009	1.19 (0.91–1.56)	0.209	12.60 (−19.88–45.07)	0.447
>2	0.84 (0.62–1.13)	0.243	1.37 (1.03–1.83)	0.031	5.92 (−30.63–42.46)	0.751
Travel time to the ART clinic (min)						
[0,15]	1.00 (Ref)		1.00 (Ref)		0.00 (Ref)	
(15,30]	0.94 (0.77–1.15)	0.571	0.98 (0.84–1.14)	0.787	31.75 (5.67–57.82)	0.017
(30,60]	0.88 (0.65–1.18)	0.391	1.03 (0.77–1.38)	0.845	31.66 (6.21–57.12)	0.015
>60	1.13 (0.82–1.54)	0.463	0.86 (0.62–1.19)	0.365	31.44 (−0.87–63.75)	0.057
Waiting time for today’s ART visit (min)						
0	1.00 (Ref)		1.00 (Ref)		0.00 (Ref)	
(0,20]	0.84 (0.62–1.15)	0.281	1.69 (1.10–2.59)	0.016	119.47 (11.20–227.75)	0.031
(20,60]	0.88 (0.56–1.38)	0.577	1.60 (1.05–2.44)	0.028	95.81 (−7.46–199.09)	0.069
>60	1.43 (0.98–2.08)	0.061	1.74 (1.16–2.62)	0.007	93.57(−11.10–198.24)	0.080

a This regression was run among all participants. The outcome was whether participants preferred ART community delivery over standard facility-based care. We used Poisson regression with a robust error structure and adjusted standard errors for clustering at the level of the healthcare facility. Each regression only had one independent variable.

b This regression was run among those participants who stated that they preferred ART community delivery over standard facility-based care. The outcome was whether participants were willing to pay for ART community delivery (regardless of the amount). We used Poisson regression with a robust error structure and adjusted standard errors for clustering at the level of the healthcare facility. Each regression only had one independent variable.

c This regression was run among those participants who stated that they preferred ART community delivery and were willing to pay for ART community delivery. The outcome was the natural logarithm of the maximum amount (in PPP$) that participants were willing to pay. We used an Ordinary Least Squares regression and adjusted standard errors for clustering at the level of the healthcare facility. Each regression only had one independent variable.

## Discussion

Only around half of ART patients living in the neighbourhoods surrounding the study facilities preferred ART community delivery over standard facility-based care, and only approximately half of these patients were willing to pay for ART community delivery. With a mean of PPP$3.61 (∼2557.43 Tanzanian Shilling or 1.14 US dollar) for one ART community delivery visit that delivers a 2-months’ supply of antiretroviral drugs, the WTP among those clients who were willing to pay for the service was relatively low, but not negligible.

In our cluster-randomized trial of ART community delivery, the only running cost of the programme was an additional payment of PPP$14 (TSh 10 000) to an HBC per ART community delivery visit (i.e. PPP$70 per client per year) to compensate them for the increased workload. Using this value as the cost of delivering ART community delivery and ignoring any cost savings to the health system from shifting care from more highly paid to lower-paid healthcare workers, the ART community delivery programme in Dar es Salaam cannot be fully financed through client contributions. In fact, even if ART community delivery was only offered to those willing to pay for the service and everyone was charged their maximum WTP, the programme could only be financed by about a quarter [25.8% (PPP$3.61/PPP$14.00)] from client contributions. To be financially viable, ART community delivery would, therefore, need to, at least in part, be financed through government or donor sources. The relatively low WTP may be partially explained by an unfamiliarity with the programme. Those who received ART community delivery in our trial were substantially more likely to state that they preferred ART community delivery and were willing to pay for the service. However, conditional on being willing to pay for the service, those who received the service in the past were, on average, not willing to pay a higher amount than those who had never received it.

Apart from full funding or subsidization through governmental and donor sources, there are two broad types of financing models for ART community delivery: one in which all patients pay the same price and only those willing to pay that price receive the service, and one in which some patients cross-subsidize the cost of the service for other patients. Under the first model, using the price that maximizes revenue for the programme, and assuming five HBC visits per year plus one facility-based check-up per year (at which patients can pick up a new 2-months’ supply of antiretroviral drugs), ART community delivery would only reach 2.3% of all eligible ART patients but raise PPP$63.55 per patient per year among this small minority of patients. Under the second model, with all those preferring ART community delivery over standard facility-based care receiving the service, the programme would reach half (48.0%) of eligible ART patients but only raise PPP$6.35 per patient per year. It could be argued that from an equity perspective, the ideal financing mechanism would demand a higher price from clients of a higher socioeconomic status to cross-subsidize the cost of the service for poorer clients. However, while they were more likely to be willing to pay for the service, those with a higher level of education (and, thus, likely a higher socioeconomic status) in our study were less likely to prefer ART community delivery over standard facility-based care and, conditional on being willing to pay for the service, there was no significant difference in the amount that more educated patients were willing to pay compared with less educated ones.

WTP can be studied from a social value (i.e. asking what is the social value of the service for society?) or a marketing (i.e. asking how much revenue can be generated from the service and what is the revenue-maximizing price point?) perspective (Kanya *et al.*, 2019). We chose a marketing perspective for this study because our research question was the degree to which ART community delivery could be financed by the service recipients. However, regardless of the WTP perspective used, this is the first study of WTP for a CHW-provided HIV care service. WTP studies have been used infrequently in healthcare research in low- and middle-income countries. In the HIV field, several studies have examined WTP for receiving ART vs not receiving ART (Muko *et al.*, 2004; Mbachu *et al.*, 2018; Tran *et al.*, 2018), one study investigated WTP for CD4-cell count and viral load monitoring (Nguyen *et al.*, 2017), and one study reported WTP for mobile-phone-based ART adherence support (Tran and Houston, 2012). The only two studies that we identified as reporting WTP for a CHW-provided service were a WTP study for a diabetes prevention programme in North Carolina (Alva *et al.*, 2017), finding that participants were willing to pay more for programmes led by registered professionals than by CHWs, and WTP for receiving injectable contraceptive depot-medroxyprogesterone acetate in Mozambique (Jacinto *et al.*, 2016), which found that 64% of women were willing to pay for the service.

This study has several limitations. First and foremost, the amount that patients stated that they were willing to pay in the questionnaire may not be the amount that they would be willing to pay if the programme was implemented in the future. It is unclear whether this limitation resulted in this study overestimating or underestimating WTP. On the one hand, participants may have wanted to appear generous (or grateful if they have received ART community delivery as part of the cluster-randomized trial and/or interacted with their interviewer before the study exit questionnaire as part of the cluster-randomized trial) to the interviewer and thus overstated the amount that they were willing to pay. Participants may also have been too optimistic in predicting future financial pressures that they may face. On the other hand, they may also have strategically under-reported their WTP anticipating that they may be asked to pay the stated amount in the future. Second, we only sampled ART patients who were residing in the neighbourhoods surrounding the healthcare facility that they were attending for ART care. It is possible that ART patients who reside further away from the facility (presumably by choice because most neighbourhoods in Dar es Salaam have an ART care facility in close proximity) had a different WTP for ART community delivery. We restricted our study to patients living in surrounding neighbourhoods because the HBCs affiliated with a healthcare facility only work in the neighbourhoods surrounding that facility. Third, our findings are specific to Dar es Salaam and may not necessarily be generalizable to other settings.

## Conclusion

It appears to be possible to raise a considerable amount of funds for CHW-led ART community delivery in Dar es Salaam from patients themselves. However, the revenue generated likely only covers a minority of the costs of such a programme and, thus, major co-financing by governments or donors would still be required. In addition, the opportunity for cross-subsidization by charging a higher price to individuals with a higher socioeconomic status likely is limited. Further to the possibility of decongesting healthcare facilities, improving adherence to antiretroviral drugs, and increasing retention in ART care, CHW-led ART community delivery offers an opportunity for cost savings for health systems because care is shifted from more highly paid health worker cadres (nurses and physicians) to a lower-paid one (CHWs). Given the rising number of individuals eligible for ART in Tanzania (Wang *et al.*, 2016), these cost savings could be large and would likely increase over time as demand for ART care increases. Along with involving patients themselves in the financing of such programmes, governments should attempt to translate these potential savings into additional funds that can be used for financing ART community delivery.

## Supplementary data

Supplementary data are available at *Health Policy and Planning* online.

## Supplementary Material

czaa088_Supplementary_Data

## References

[czaa088-B1] Alva ML , Samuel-Hodge CD, Porterfield D, Thomas T, Leeman J. 2017 A feasibility study of supply and demand for diabetes prevention programs in North Carolina. Preventing Chronic Disease 14: E51. 28662760 10.5888/pcd14.160604 PMC5494814

[czaa088-B2] Barennes H , Frichittavong A, Gripenberg M, Koffi P. 2015 Evidence of high out of pocket spending for HIV care leading to catastrophic expenditure for affected patients in Lao people's democratic republic. PLoS One 10: e0136664. 26327558 10.1371/journal.pone.0136664 PMC4556637

[czaa088-B3] Chimbindi N , Bor J, Newell ML et al 2015 Time and money: the true costs of health care utilization for patients receiving ‘free’ HIV/TB care and treatment in rural KwaZulu-Natal. *Journal of Acquired Immune Deficiency Syndromes* 70: e52-e60. 10.1097/QAI.0000000000000728 PMC4748708 26371611

[czaa088-B55365537] Di Giorgio L Moses MW Fullman N et al 2016 The potential to expandantiretroviral therapy by improving health facility efficiency: evidence from Kenya, Uganda, and Zambia. BMC Medicine 14: 108. 10.1186/s12916-016-0653-z PMC4952151 27439621

[czaa088-B4] Etiaba E , Onwujekwe O, Torpey K, Uzochukwu B, Chiegil R. 2016 What is the economic burden of subsidized HIV/AIDS treatment services on patients in Nigeria and is this burden catastrophic to households? PLoS One 11: e0167117. 27911921 10.1371/journal.pone.0167117 PMC5135056

[czaa088-B2529807] Gallis JA Turner EL. 2019 Relative measures of association for binary outcomes: challenges and recommendations for the global health researcher [published correction appears in Ann Glob Health. 2020 Jul 06;86(1):77]. AnnGlob Health 85: 137. 10.5334/aogh.2581 PMC6873895 31807416

[czaa088-B5] Geldsetzer P , Fink G, Vaikath M, Barnighausen T. 2018a. Sampling for patient exit interviews: assessment of methods using mathematical derivation and computer simulations. Health Services Research 53: 256–72. 27882543 10.1111/1475-6773.12611 PMC5785309

[czaa088-B6] Geldsetzer P , Francis JM, Sando D et al 2018b. Community delivery of antiretroviral drugs: a non-inferiority cluster-randomized pragmatic trial in Dar es Salaam, Tanzania. PLoS Medicine 15: e1002659. 30231024 10.1371/journal.pmed.1002659 PMC6145501

[czaa088-B7] Geldsetzer P , Francis JM, Ulenga N et al 2017 The impact of community health worker-led home delivery of antiretroviral therapy on virological suppression: a non-inferiority cluster-randomized health systems trial in Dar es Salaam. BMC Health Services Research 17: 160. 28228134 10.1186/s12913-017-2032-7 PMC5322683

[czaa088-B8] Geldsetzer P , Ortblad K, Barnighausen T. 2016 The efficiency of chronic disease care in sub-Saharan Africa. BMC Medicine 14: 127. 27566531 10.1186/s12916-016-0675-6 PMC5002156

[czaa088-B9] Global Burden of Disease Health Financing Collaborator Network. 2018 Spending on health and HIV/AIDS: domestic health spending and development assistance in 188 countries. Lancet 391: 1799–829. 29678342 10.1016/S0140-6736(18)30698-6 PMC5946845

[czaa088-B10] Hontelez JA , de Vlas SJ, Baltussen R et al 2012 The impact of antiretroviral treatment on the age composition of the HIV epidemic in sub-Saharan Africa. AIDS 26(Suppl): S19–30. 22781175 10.1097/QAD.0b013e3283558526 PMC3886374

[czaa088-B11] Jacinto A , Mobaracaly MR, Ustab MB et al 2016 Safety and acceptability of community-based distribution of injectable contraceptives: a pilot project in Mozambique. Global Health: Science and Practice 4: 410–21. 10.9745/GHSP-D-16-00133 PMC5042697 27651076

[czaa088-B12] Jaffar S , Amuron B, Foster S et al 2009 Rates of virological failure in patients treated in a home-based versus a facility-based HIV-care model in Jinja, southeast Uganda: a cluster-randomised equivalence trial. The Lancet 374: 2080–9. 10.1016/S0140-6736(09)61674-3 PMC2806484 19939445

[czaa088-B13] Kanya L , Sanghera S, Lewin A, Fox-Rushby J. 2019 The criterion validity of willingness to pay methods: a systematic review and meta-analysis of the evidence. Social Science & Medicine 232: 238–61. 31108330 10.1016/j.socscimed.2019.04.015

[czaa088-B14] Klose T. 1999 The contingent valuation method in health care. Health Policy 47: 97–123. 10538292 10.1016/s0168-8510(99)00010-x

[czaa088-B15] Lazarus JV , Safreed-Harmon K, Nicholson J, Jaffar S. 2014 Health service delivery models for the provision of antiretroviral therapy in sub-Saharan Africa: a systematic review. Tropical Medicine & International Health 19: 1198–215. 25065882 10.1111/tmi.12366

[czaa088-B16] Mbachu C , Okoli C, Onwujekwe O, Enabulele F. 2018 Willingness to pay for antiretroviral drugs among HIV and AIDS clients in south-east Nigeria. Health Expectations 21: 270–8. 28805985 10.1111/hex.12612 PMC5750729

[czaa088-B17] Mitchell R , Carson R. 1984. A contingent valuation estimate of national freshwater benefits: technical report to the U.S. Environmental Protection Agency. Washington, D.C: Resources for the Future.

[czaa088-B18] Mitchell R , Carson R. 1993. Using surveys to value public goods: the contingent valuation method. Washington, D.C: Resources for the Future.

[czaa088-B19] Muko KN , Ngwa VC, Chigang LC et al 2004 Willingness to pay for treatment with highly active antiretroviral (HAART) drugs: a rural case study in Cameroon. Sahara-J: Journal of Social Aspects of HIV/AIDS 1: 107–13. 10.1080/17290376.2004.9724833 17601016

[czaa088-B20] Nguyen QL , Nguyen LH, Tran BX et al 2017 Co-financing for viral load monitoring during the course of antiretroviral therapy among patients with HIV/AIDS in Vietnam: a contingent valuation survey. PLoS One 12: e0172050. 28199405 10.1371/journal.pone.0172050 PMC5310871

[czaa088-B21] Phillips A , Shroufi A, Vojnov L et al 2015 Sustainable HIV treatment in Africa through viral-load-informed differentiated care. Nature 528: S68–76. 26633768 10.1038/nature16046 PMC4932825

[czaa088-B22] Ryan M , Scott DA, Donaldson C. 2004 Valuing health care using willingness to pay: a comparison of the payment card and dichotomous choice methods. Journal of Health Economics 23: 237–58. 15019754 10.1016/j.jhealeco.2003.09.003

[czaa088-B23] Ryan M , Scott DA, Reeves C et al 2001 Eliciting public preferences for healthcare: a systematic review of techniques. Health Technology Assessment 5: 1–186. 10.3310/hta5050 11262422

[czaa088-B24] Selke HM , Kimaiyo S, Sidle JE et al 2010 Task-shifting of antiretroviral delivery from health care workers to persons living with HIV/AIDS: clinical outcomes of a community-based program in Kenya. Journal of Acquired Immune Deficiency Syndromes 55: 483–90. 20683336 10.1097/QAI.0b013e3181eb5edb

[czaa088-B25] Tran BX , Fleming M, Nguyen CT, Latkin CA. 2018 Financial mobilization for antiretroviral therapy program: multi-level predictors of willingness to pay among patients with HIV/AIDS in Vietnam. AIDS Care 30: 1488–97. 30047280 10.1080/09540121.2018.1503633

[czaa088-B26] Tran BX , Houston S. 2012 Mobile phone-based antiretroviral adherence support in Vietnam: feasibility, patient's preference, and willingness-to-pay. IDS and Behavior 16: 1988–92. 10.1007/s10461-012-0271-5 22814571

[czaa088-B27] UNAIDS. 2019 United Republic of Tanzania. Geneva, Switzerland https://www.unaids.org/en/regionscountries/countries/unitedrepublicoftanzania, accessed July 2020.

[czaa088-B28] United Nations. 2016 The World’s Cities in 2016. New York: United Nations.

[czaa088-B29] Waldrop G , Doherty M, Vitoria M, Ford N. 2016 Stable patients and patients with advanced disease: consensus definitions to support sustained scale up of antiretroviral therapy. Tropical Medicine & International Health 21: 1124–30. 27371814 10.1111/tmi.12746

[czaa088-B30] Wang H , Wolock TM, Carter A et al 2016 Estimates of global, regional, and national incidence, prevalence, and mortality of HIV, 1980-2015: the Global Burden of Disease Study 2015. The Lancet HIV 3: e361–87. 27470028 10.1016/S2352-3018(16)30087-X PMC5056319

[czaa088-B31] World Health Organization. 2016 Consolidated Guidelines on the Use of Antiretroviral Drugs for Treating and Preventing HIV infection—Recommendations for a Public Health Approach. World Health Organization 1. 27466667

[czaa088-B32] World Health Organisation. 2018 World Health Statistics 2018: Monitoring Health for the SDGs. World Health Organisation.

[czaa088-B33] Zou G. 2004 A modified Poisson regression approach to prospective studies with binary data. American Journal of Epidemiology 159: 702–6. 15033648 10.1093/aje/kwh090

