# Supplementary material for: Willingness to pay for community delivery of antiretroviral treatment in urban Tanzania: a cross-sectional survey
Source: Health Policy Plan. 2020 Oct 23;35(10):1300–8. doi: 10.1093/heapol/czaa088 (PMC7886440; doi:10.1093/heapol/czaa088)
Supplement: czaa088_Supplementary_Data [file czaa088_supplementary_data.zip › Appendix_2020-06-18_clean.docx]

**Supplementary appendix**

Text S1. Questions used to ascertain willingness to pay for ART community delivery 2

Figure S1. Willingness to pay for one community delivery of a two-months’ supply of antiretroviral drugs among participants preferring ART community delivery over standard facility-based care, stratified by whether participants had received ART community delivery before 4

Table S1. Willingness to pay for one community delivery of a two-months’ supply of antiretroviral drugs (PPP$) 5

Table S2. Covariate-adjusted regressions for predictors of preferring ART community delivery over standard facility-based care and willingness to pay 6

# **Text S1. Questions used to ascertain willingness to pay for ART community delivery**

| **5.1** As explained earlier, a home-based carer is someone from the community who regularly visits households. Home-based carers provide information on how to stay healthy and help care for ill people at their home.  If you had the option to freely choose among the following two ways of receiving your ARVs, which one would you prefer?  **Option 1:** Delivery of ARVs to your home or somewhere else in your community every 2 months by a home-based carer. In addition, you have to come to this healthcare facility every 6 months for a check-up.  **Option 2:** Attending this healthcare facility every 2 months to get your ARVs.  In Option 1 you have to come to this healthcare facility every 6 months. In option 2, you have to come to this healthcare facility every 2 months.  Which option do you prefer?  *Please circle a number.* | Option 1: ARV delivery through a HBC ........................... | | | 1 |
| --- | --- | --- | --- | --- |
|  | Option 2: Attending the healthcare facility every 2 months (**skip to Q6.1**)…………………………………… | | | 2 |
|  |  | | |  |
| **5.2**  The following few questions are all imaginary scenarios for research purposes only. None of your answers to these questions will affect your care in any way or whether you have to pay for it.  Please consider the following hypothetical choice: Assume that you have to pay your home-based carer money to deliver your ARVs to your home or somewhere else in your community every two months. The alternative is to come to this healthcare facility every two months to pick up your ARVs, which is free of charge. Would you be willing to pay your home-based carer to deliver your ARVs to you?  Yes ……………………..1  No (**skip to Q6.1**)………2  **5.3** When answering the next question, please bear in mind what you can actually afford to pay or feasibly borrow. How much money would you be willing to pay your home-based carer every two months to deliver ARVs to you? The alternative is to come to this healthcare facility every two months to pick up your ARVs, which is free of charge.  Please answer yes or no for each of the following amounts. *Instruction to interviewer:* *Ask* *“Would you be willing to pay the HBC [amount]?” and then note down ‘yes’ or ‘no’. As soon as the respondent says “no” to an amount, you can stop and move on to the next question.* | | | | |
|  | **Yes** | **No** |  | |
| 1-10 TZS | □ | □ |  | |
| 11-20 TZS | □ | □ |  | |
| 21-40 TZS | □ | □ |  | |
| 41-60 TZS | □ | □ |  | |
| 61-80 TZS | □ | □ |  | |
| 81-100 TZS | □ | □ |  | |
| 101-200 TZS | □ | □ |  | |
| 201-400 TZS | □ | □ |  | |
| 401-600 TZS | □ | □ |  | |
| 601-800 TZS | □ | □ |  | |
| 801-1,000 TZS | □ | □ |  | |
| 1,001-2,000 TZS | □ | □ |  | |
| 2,001-4,000 TZS | □ | □ |  | |
| 4,001-6,000 TZS | □ | □ |  | |
| 6,001-8,000 TZS | □ | □ |  | |
| 8,001-10,000 TZS | □ | □ |  | |
| 10,001-20,000 TZS | □ | □ |  | |
| 20,001-40,000 TZS | □ | □ |  | |
| 40,001-60,000 TZS | □ | □ |  | |
| 60,001-80,000 TZS | □ | □ |  | |
| 80,001-100,000 TZS | □ | □ |  | |
| 100,001-200,000 TZS | □ | □ |  | |
| 200,001-400,000 TZS | □ | □ |  | |
| 400,001-600,000 TZS | □ | □ |  | |
| 600,001-800,000 TZS | □ | □ |  | |
| 800,001-1,000,000 TZS | □ | □ |  | |
| 1,000,001-2,000,000 TZS | □ | □ |  | |
| 2,000,001-4,000,000 TZS | □ | □ |  | |
| 4,000,001-6,000,000 TZS | □ | □ |  | |
| 6,000,001-8,000,000 TZS | □ | □ |  | |
| 8,000,001-10,000,000 TZS | □ | □ |  | |

Figure S1. Willingness to pay for one community delivery of a two-months’ supply of antiretroviral drugs among participants preferring ART community delivery over standard facility-based care, stratified by whether participants had received ART **at home** before**^1^**

^^

^1^ The x-axis is on a logarithmic scale.

PPP$=purchasing-power-parity-adjusted dollars

# **Table S1. Willingness to pay for one community delivery of a two-months’ supply of antiretroviral drugs (PPP$)**

|  | All participants | Participants preferring ART community delivery | Participants willing to pay for ART community delivery |
| --- | --- | --- | --- |
| Mean (SD) | 0.84 (3.60) | 1.86 (5.18) | 3.61 (6.77) |
| Median (IQR) | 0.00 (0.00) | 0.42 (2.12) | 1.27 (0.85) |

# **Table S2. Covariate-adjusted regressions for predictors of preferring ART community delivery over standard facility-based care and willingness to pay**

|  | ***Preferring ART community delivery^1^*** | | ***Willing to pay for ART community delivery^2^*** | | ***Amount willing to pay^3^*** | |
| --- | --- | --- | --- | --- | --- | --- |
|  | ***RR (95% CI)*** | ***P*** | ***RR (95% CI)*** | ***P*** | ***% change (95% CI)*** | ***P*** |
| Sex |  |  | 1.00 (Ref)  1.11 (0.90 - 1.36) |  | 0.00 (Ref)  12.29 (-12.81 - 37.39) |  |
| Female | 1.00 (Ref)  1.02 (0.91 - 1.15) |  |  |  |  |  |
| Male |  | 0.724 |  | 0.339 |  | 0.337 |
| Age (years) |  |  | 1.00 (Ref)  1.03 (0.81 - 1.32)  0.76 (0.59 - 0.99)  0.68 (0.50 - 0.94) |  | 0.00 (Ref)  -2.24 (-43.49 - 39.00)  9.76 (-21.88 - 41.39)  -14.53 (-54.66 - 25.60) |  |
| [18,28] | 1.00 (Ref)  0.93 (0.76 - 1.13)  0.90 (0.70 - 1.16)  0.95 (0.73 - 1.23) |  |  |  |  |  |
| (28,38] |  | 0.455 |  | 0.811 |  | 0.915 |
| (38,48] |  | 0.413 |  | 0.045 |  | 0.546 |
| > 48 |  | 0.693 |  | 0.018 |  | 0.478 |
| Education | 1.00 (Ref) |  | 1.00 (Ref)  1.02 (0.80 - 1.30)  1.33 (1.01 - 1.76) |  | 0.00 (Ref)  -3.71 (-44.07 - 36.66)  22.07 (-14.82 - 58.95) |  |
| None |  |  |  |  |  |  |
| Primary School | 0.80 (0.69 - 0.92)  0.77 (0.60 - 1.00) | 0.002 |  | 0.872 |  | 0.857 |
| Secondary school or above |  | 0.046 |  | 0.046 |  | 0.241 |
| Marital Status |  |  | 1.00 (Ref)  1.20 (1.07 - 1.33) |  | 0.00 (Ref)  1.82 (-22.33 - 25.98) |  |
| Not married | 1.00 (Ref) |  |  |  |  |  |
| Married | 1.04 (0.91 - 1.19) | 0.533 |  | 0.001 |  | 0.882 |
| Years since initiation of ART | 1.00 (Ref)  1.07 (0.94 - 1.21)  1.01 (0.86 - 1.18) |  | 1.00 (Ref)  0.97 (0.80 - 1.17)  1.14 (0.92 - 1.41) |  | 0.00 (Ref)  20.67 (-4.36 - 45.71)  -11.31 (-46.96 - 24.35) |  |
| [0,3] |  |  |  |  |  |  |
| (3,5] |  | 0.303 |  | 0.733 |  | 0.106 |
| > 5 |  | 0.916 |  | 0.220 |  | 0.534 |
| Mode of ART community delivery | 1.00 (Ref)  1.40 (1.05 - 1.87)  1.57 (1.20 - 2.04) |  | 1.00 (Ref)  1.31 (0.96 - 1.78)  1.51 (1.03 - 2.20) |  | 0.00 (Ref)  -49.67 (-109.72 - 10.37)  -40.40 (-122.20 - 41.39) |  |
| Once a month |  |  |  |  |  |  |
| Every two months |  | 0.021 |  | 0.085 |  | 0.105 |
| Brought home |  | 0.001 |  | 0.033 |  | 0.333 |
| Disclosed HIV status | 1.00 (Ref)  0.76 (0.61 - 0.94) |  | 1.00 (Ref)  0.69 (0.45 - 1.07) |  | 0.00 (Ref)  5.64 (-39.99 - 51.28) |  |
| Yes |  |  |  |  |  |  |
| No |  | 0.013 |  | 0.097 |  | 0.808 |
| Received ART community delivery | 1.00 (Ref)  2.50 (1.82 - 3.42) |  | 1.00 (Ref)  0.89 (0.74 - 1.09) |  | 0.00 (Ref)  -14.68 (-57.75 - 28.38) |  |
| No |  |  |  |  |  |  |
| Yes |  | 0.000 |  | 0.266 |  | 0.504 |
| Total costs for today’s ART visit (PPP$) | 1.00 (Ref)  0.95 (0.73 - 1.22)  0.72 (0.53 - 0.98)  0.95 (0.74 - 1.22) |  | 1.00 (Ref)  1.17 (0.94 - 1.46)  1.11 (0.89 - 1.38)  1.39 (1.10 - 1.75) |  | 0.00 (Ref)  16.17 (-13.37 - 45.73)  -9.50 (-45.96 - 26.96)  0.77 (-36.66 - 38.21) |  |
| 0 |  |  |  |  |  |  |
| (0,1] |  | 0.673 |  | 0.153 |  | 0.283 |
| (1,2] |  | 0.038 |  | 0.370 |  | 0.610 |
| > 2 |  | 0.686 |  | 0.006 |  | 0.968 |
| Travel time to the ART clinic (minutes) | 1.00 (Ref)  1.03 (0.88 - 1.20)  0.97 (0.77 - 1.21)  1.09 (0.85 - 1.40) |  | 1.00 (Ref)  0.98 (0.81 - 1.18)  0.95 (0.73 - 1.24)  0.90 (0.63 - 1.28) |  | 0.00 (Ref)  35.24 (11.52 - 58.96)  38.75 (7.57 - 69.94)  35.50 (-11.67 - 82.68) |  |
| [0,15] |  |  |  |  |  |  |
| (15,30] |  | 0.710 |  | 0.791 |  | 0.004 |
| (30,60] |  | 0.763 |  | 0.710 |  | 0.015 |
| > 60 |  | 0.493 |  | 0.546 |  | 0.140 |
| Waiting time for today’s ART visit (minutes) | 1.00 (Ref)  1.20 (0.93 - 1.56)  1.17 (0.89 - 1.54)  1.48 (1.15 - 1.90) |  | 1.00 (Ref)  1.75 (1.09 - 2.79)  1.65 (1.07 - 2.54)  1.72 (1.09 - 2.70) |  | 0.00 (Ref)  94.80 (-18.15 - 207.76)  78.74 (-34.65 - 192.15)  81.29 (-36.44 - 199.03) |  |
| 0 |  |  |  |  |  |  |
| (0,20] |  | 0.167 |  | 0.019 |  | 0.100 |
| (20,60] |  | 0.255 |  | 0.025 |  | 0.173 |
| > 60 |  | 0.002 |  | 0.019 |  | 0.176 |

^1^ This regression was run among all participants. The outcome was whether participants preferred ART community delivery over standard facility-based care. We used Poisson regression with a robust error structure and adjusted standard errors for clustering at the level of the healthcare facility. The regression had all variables shown in the table as independent variables.

^2^ This regression was run among those participants who stated that they preferred ART community delivery over standard facility-based care. The outcome was whether participants were willing to pay for ART community delivery (regardless of the amount). We used Poisson regression with a robust error structure and adjusted standard errors for clustering at the level of the healthcare facility. The regression had all variables shown in the table as independent variables.

^3^ This regression was run among those participants who stated that they preferred ART community delivery and were willing to pay for ART community delivery. The outcome was the natural logarithm of the maximum amount (in PPP$) that participants were willing to pay. We used an Ordinary Least Squares regression and adjusted standard errors for clustering at the level of the healthcare facility.
