# Supplementary material for: Willingness to pay for community delivery of antiretroviral treatment in urban Tanzania: a cross-sectional survey
Source: Health Policy Plan. 2020 Oct 23;35(10):1300–8. doi: 10.1093/heapol/czaa088 (PMC7886440; doi:10.1093/heapol/czaa088)
Supplement: czaa088_Supplementary_Data [file czaa088_supplementary_data.zip › Figure1_2020-01-16.docx]

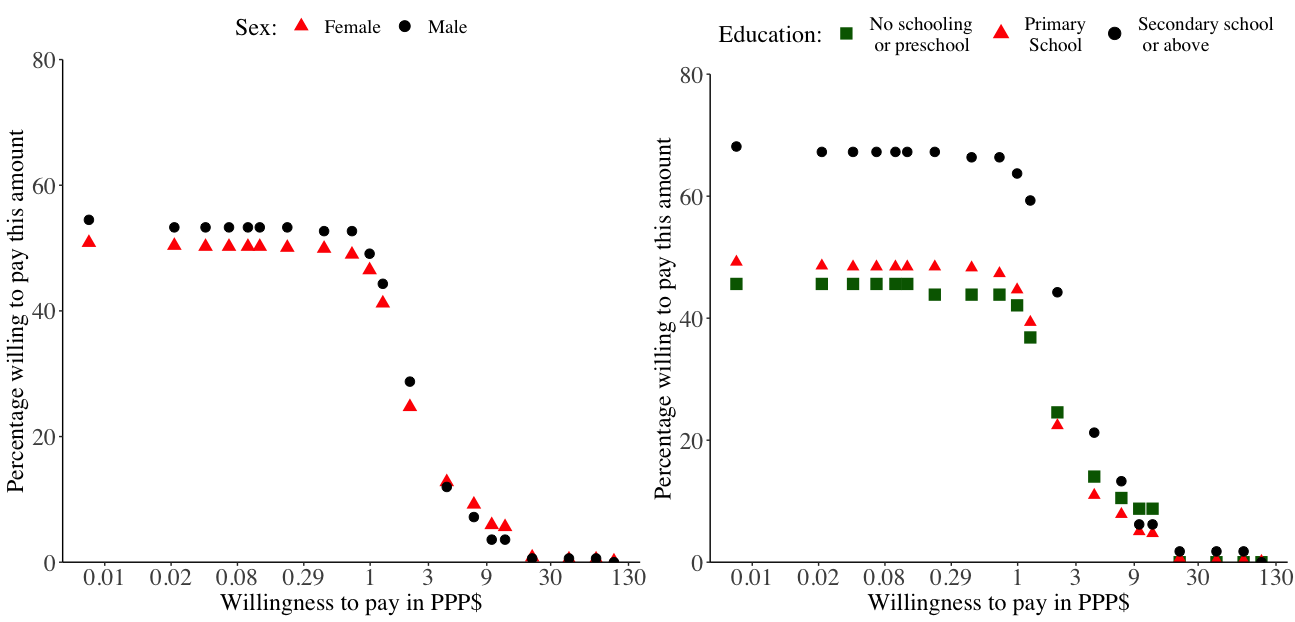


**Figure 1. Willingness to pay for one community delivery of a two-months’ supply of antiretroviral drugs among participants preferring ART community delivery over standard facility-based care (n=810)^1^**

^1^ The x-axis is on a logarithmic scale.

PPP$=purchasing-power-parity-adjusted dollars
