# Supplementary material for: Willingness to pay for community delivery of antiretroviral treatment in urban Tanzania: a cross-sectional survey
Source: Health Policy Plan. 2020 Oct 23;35(10):1300–8. doi: 10.1093/heapol/czaa088 (PMC7886440; doi:10.1093/heapol/czaa088)
Supplement: czaa088_Supplementary_Data [file czaa088_supplementary_data.zip › Figure2_2020-01-16.docx]

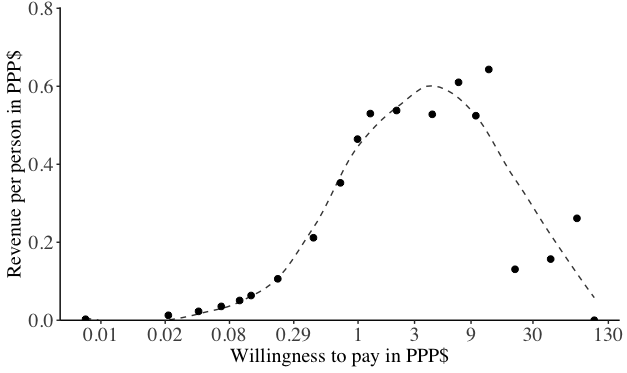


**Figure 2. Total revenue generated per person by one community delivery of a two-months’ supply of antiretroviral drugs^1,2,3^**

^1^ This figure refers to total revenue per person among those participants who preferred ARV community delivery over standard facility-based care.

^2^ The x-axis is on a logarithmic scale.

^3^ The dashed line represents a LOESS regression with a bandwidth of 0.5.

PPP$=purchasing-power-parity-adjusted dollars
