# Supplementary material for: Willingness to pay for community delivery of antiretroviral treatment in urban Tanzania: a cross-sectional survey
Source: Health Policy Plan. 2020 Oct 23;35(10):1300–8. doi: 10.1093/heapol/czaa088 (PMC7886440; doi:10.1093/heapol/czaa088)
Supplement: czaa088_Supplementary_Data [file czaa088_supplementary_data.zip › Table1_2020-06-18_clean.docx]

*Table 1. Sample characteristics*

|  | All participants | Preferring ART  community delivery |
| --- | --- | --- |
| N | 1799 | 810 |
| Female^1,2^ | 1464 (81.4%) | 643 (79.4%) |
| Age (years)^1^ |  |  |
| 18-28 | 153 (8.5%) | 59 (7.3%) |
| 29-38 | 651 (36.2%) | 273 (33.7%) |
| 39-48 | 685 (38.1%) | 322 (39.8%) |
| > 48 | 304 (16.9%) | 154 (19%) |
| Highest level of school attended |  |  |
| No schooling or preschool | 87 (4.8%) | 57 (7%) |
| Primary School | 1418 (78.8%) | 638 (78.8%) |
| Secondary School or above | 285 (15.8%) | 113 (14%) |
| *Missing* | *9 (0.5%)* | *2 (0.2%)* |
| Currently married^2^ | 755 (42.0%) | 354 (43.7%) |
| *Missing* | *21 (1.2%)* | *4 (0.5%)* |
| Disclosed HIV status to at least one person^2^ | 1630 (90.6%) | 757 (93.5%) |
| *Missing* | *4 (0.2%)* | *1 (0.1%)* |
| Received ART community delivery^1,2^ | 388 (21.6%) | 362 (44.7%) |
| Mode of antiretroviral drug refills |  |  |
| Once a month at the facility | 474 (26.3%) | 137 (16.9%) |
| Every two months at the facility | 1200 (66.7%) | 564 (69.6%) |
| Brought to the patient by an HBC^3^ | 102 (5.7%) | 100 (12.3%) |
| *Missing* | *23 (1.3%)* | *9 (1.1%)* |
| Total cost for today’s ART visit (PPP$)^1^ |  |  |
| 0.00 | 661 (36.7%) | 341 (42.1%) |
| 0.01-1.00 | 602 (33.5%) | 276 (34.1%) |
| 1.01-2.00 | 367 (20.4%) | 120 (14.8%) |
| > 2.00 | 169 (9.4%) | 73 (9.0%) |
| Waiting time for today’s ART visit (minutes) |  |  |
| 0 | 225 (12.5%) | 104 (12.8%) |
| 1-20 | 780 (43.4%) | 304 (37.5%) |
| 21-60 | 465 (25.8%) | 189 (23.3%) |
| > 60 | 271 (15.1%) | 179 (22.1%) |
| *Missing* | *58 (3.2%)* | *34 (4.2%)* |
| Travel time to the ART clinic (minutes) |  |  |
| 0-15 | 600 (33.4%) | 281 (34.7%) |
| 16-30 | 817 (45.4%) | 361 (44.6%) |
| 31-60 | 285 (15.8%) | 117 (14.4%) |
| > 60 | 93 (5.2%) | 49 (6.0%) |
| *Missing* | *4 (0.2%)* | *2 (0.2%)* |
| Number of HBC visits during the study period^1^ |  |  |
| 0 | 1400 (77.8%) | 450 (55.6%) |
| 1-3 | 205 (11.4%) | 171 (21.1%) |
| > 3 | 194 (10.8%) | 189 (23.3%) |
| Years since initiation of ART |  |  |
| 0-3 | 747 (41.5%) | 286 (35.3%) |
| 4-5 | 467 (26.0%) | 222 (27.4%) |
| > 5 | 489 (27.2%) | 248 (30.6%) |
| *Missing* | *96 (5.3%)* | *54 (6.7%)* |

Abbreviations: PPP$=purchasing-power-parity-adjusted dollars; ART=antiretroviral therapy; HBC=home-based carer

^1^ No observations were missing for these variables.

^2^ These are binary variables. We show the number and percent for participants who answered “yes” to this variable (i.e., those who were female rather than male, those who were currently married rather than currently not married, those who disclosed their HIV status to at least one person rather than those who did not disclose their status to anyone, and those who received ART community delivery rather than those who did not receive ART community delivery).

^3^ We suspect that fewer participants answered that they received their ARVs at home through an HBC for this question than for the question on having received ART community delivery because they misunderstood the question to refer to the frequency of antiretroviral drug refills rather than the mode of the refills (picking up antiretroviral drugs at the healthcare facility versus receiving them at home).
