# Supplementary material for: Willingness to pay for community delivery of antiretroviral treatment in urban Tanzania: a cross-sectional survey
Source: Health Policy Plan. 2020 Oct 23;35(10):1300–8. doi: 10.1093/heapol/czaa088 (PMC7886440; doi:10.1093/heapol/czaa088)
Supplement: czaa088_Supplementary_Data [file czaa088_supplementary_data.zip › Table2_2020-06-18_clean.docx]

*Table 2. Predictors of willingness to pay and preferring ART community delivery over standard facility-based care*

|  | ***Preferring ART community delivery^1^*** | | | ***Willing to pay for ART community delivery^2^*** | | | ***Amount willing to pay^3^*** | | |
| --- | --- | --- | --- | --- | --- | --- | --- | --- | --- |
|  | ***RR (95% CI)*** | | ***P*** | ***RR (95% CI)*** | | ***P*** | ***% change (95% CI)*** | | ***P*** |
| Sex |  |  |  |  |  |  |  |  |  |
| Female | 1.00 (Ref) |  |  | 1.00 (Ref) |  |  | 0.00 (Ref) |  |  |
| Male | 1.14 (0.97 - 1.33) | | 0.122 | 1.07 (0.88 - 1.30) | | 0.506 | -8.44 (-38.81- 21.94) | | 0.586 |
| Age (years) |  |  |  |  |  |  |  |  |  |
| [18,28] | 1.00 (Ref) |  |  | 1.00 (Ref) |  |  | 0.00 (Ref) |  |  |
| (28,38] | 1.09 (0.88 - 1.34) | | 0.433 | 0.98 (0.78 - 1.24) | | 0.895 | -15.49 (-51.32 - 20.33) | | 0.397 |
| (38,48] | 1.22 (0.93 - 1.59) | | 0.144 | 0.78 (0.62 - 0.99) | | 0.042 | -15.78 (-45.71 - 14.14) | | 0.301 |
| > 48 | 1.31 (0.97 - 1.77) | | 0.074 | 0.69 (0.51 - 0.93) | | 0.015 | -33.65 (-77.38 - 10.09) | | 0.132 |
| Education |  |  |  |  |  |  |  |  |  |
| None | 1.00 (Ref) |  |  | 1.00 (Ref) |  |  | 0.00 (Ref) |  |  |
| Primary School | 0.69 (0.56 - 0.85) | | <0.001 | 1.08 (0.77 - 1.53) | | 0.651 | -21.14 (-74.38 - 32.10) | | 0.436 |
| Secondary school or above | 0.61 (0.43 - 0.86) | | 0.005 | 1.49 (1.00 - 2.22) | | 0.048 | 3.99 (-52.61 - 60.59) | | 0.890 |
| Marital Status |  |  |  |  |  |  |  |  |  |
| Not married | 1.00 (Ref) |  |  | 1.00 (Ref) |  |  | 0.00 (Ref) |  |  |
| Married | 1.06 (0.93 - 1.21) | | 0.370 | 1.19 (1.06 - 1.33) | | 0.003 | 4.87 (-24.41 - 34.14) | | 0.745 |
| Years since initiation of ART |  |  |  |  |  |  |  |  |  |
| [0,3] | 1.00 (Ref) |  |  | 1.00 (Ref) |  |  | 0.00 (Ref) |  |  |
| (3,5] | 1.24 (1.04 - 1.49) | | 0.018 | 0.98 (0.81 - 1.19) | | 0.844 | 21.46 (-3.46 - 46.37) | | 0.091 |
| > 5 | 1.32 (1.02 - 1.72) | | 0.036 | 1.07 (0.85 - 1.35) | | 0.573 | -10.79 (-36.90 - 15.33) | | 0.418 |
| Mode of ART provision |  |  |  |  |  |  |  |  |  |
| Once a month | 1.00 (Ref) |  |  | 1.00 (Ref) |  |  | 0.00 (Ref) |  |  |
| Every two months | 1.63 (1.18 - 2.24) | | 0.003 | 1.22 (0.92 - 1.61) | | 0.160 | -49.68 (-120.38 - 21.03) | | 0.168 |
| Brought home | 3.39 (2.53 - 4.55) | | <0.001 | 1.21 (0.75 - 1.94) | | 0.429 | -61.70 (-134.42 - 11.02) | | 0.096 |
| Disclosed HIV status |  |  |  |  |  |  |  |  |  |
| Yes | 1.00 (Ref) |  |  | 1.00 (Ref) |  |  | 0.00 (Ref) |  |  |
| No | 0.68 (0.52 - 0.89) | | 0.005 | 0.66 (0.43 - 1.00) | | 0.049 | 20.79 (-10.07 - 51.65) | | 0.187 |
| Received ART community delivery |  |  |  |  |  |  |  |  |  |
| No | 1.00 (Ref) |  |  | 1.00 (Ref) |  |  | 0.00 (Ref) |  |  |
| Yes | 2.94 (2.21 - 3.91) | | <0.001 | 0.90 (0.69 - 1.17) | | 0.424 | -28.77 (-72.36 - 14.82) | | 0.196 |
| Total costs for today’s ART visit (PPP$) |  |  |  |  |  |  |  |  |  |
| 0 | 1.00 (Ref) |  |  | 1.00 (Ref) |  |  | 0.00 (Ref) |  |  |
| (0,1] | 0.89 (0.61 - 1.30) | | 0.545 | 1.27 (1.00 - 1.61) | | 0.050 | 26.99 (-2.66 - 56.64) | | 0.074 |
| (1,2] | 0.63 (0.45 - 0.89) | | 0.009 | 1.19 (0.91 - 1.56) | | 0.209 | 12.60 (-19.88 - 45.07) | | 0.447 |
| > 2 | 0.84 (0.62 - 1.13) | | 0.243 | 1.37 (1.03 - 1.83) | | 0.031 | 5.92 (-30.63 - 42.46) | | 0.751 |
| Travel time to the ART clinic (minutes) |  |  |  |  |  |  |  |  |  |
| [0,15] | 1.00 (Ref) |  |  | 1.00 (Ref) |  |  | 0.00 (Ref) |  |  |
| (15,30] | 0.94 (0.77 - 1.15) | | 0.571 | 0.98 (0.84 - 1.14) | | 0.787 | 31.75 (5.67 - 57.82) | | 0.017 |
| (30,60] | 0.88 (0.65 - 1.18) | | 0.391 | 1.03 (0.77 - 1.38) | | 0.845 | 31.66 (6.21 - 57.12) | | 0.015 |
| > 60 | 1.13 (0.82 - 1.54) | | 0.463 | 0.86 (0.62 - 1.19) | | 0.365 | 31.44 (-0.87 - 63.75) | | 0.057 |
| Waiting time for today’s ART visit (minutes) |  |  |  |  |  |  |  |  |  |
| 0 | 1.00 (Ref) |  |  | 1.00 (Ref) |  |  | 0.00 (Ref) |  |  |
| (0,20] | 0.84 (0.62 - 1.15) | | 0.281 | 1.69 (1.10 - 2.59) | | 0.016 | 119.47 (11.20 - 227.75) | | 0.031 |
| (20,60] | 0.88 (0.56 - 1.38) | | 0.577 | 1.60 (1.05 - 2.44) | | 0.028 | 95.81 (-7.46 - 199.09) | | 0.069 |
| > 60 | 1.43 (0.98 - 2.08) | | 0.061 | 1.74 (1.16 - 2.62) | | 0.007 | 93.57(-11.10 - 198.24) | | 0.080 |

^1^ This regression was run among all participants. The outcome was whether participants preferred ART community delivery over standard facility-based care. We used Poisson regression with a robust error structure and adjusted standard errors for clustering at the level of the healthcare facility. Each regression only had one independent variable.

^2^ This regression was run among those participants who stated that they preferred ART community delivery over standard facility-based care. The outcome was whether participants were willing to pay for ART community delivery (regardless of the amount). We used Poisson regression with a robust error structure and adjusted standard errors for clustering at the level of the healthcare facility. Each regression only had one independent variable.

^3^ This regression was run among those participants who stated that they preferred ART community delivery and were willing to pay for ART community delivery. The outcome was the natural logarithm of the maximum amount (in PPP$) that participants were willing to pay. We used an Ordinary Least Squares regression and adjusted standard errors for clustering at the level of the healthcare facility. Each regression only had one independent variable.
